# Editorial: Eye health: challenges and solutions

**DOI:** 10.3389/fpubh.2024.1488423

**Published:** 2024-09-20

**Authors:** Xiaohui Yang, Kai Cao, Jie Hao, Mayinuer Yusufu

**Affiliations:** ^1^Department of Ophthalmology, Beijing Institute of Ophthalmology, Beijing Tongren Hospital, Capital Medical University, Beijing, China; ^2^Centre for Eye Research Australia, Royal Victorian Eye and Ear Hospital, East Melbourne, VIC, Australia; ^3^Department of Surgery (Ophthalmology), The University of Melbourne, Melbourne, VIC, Australia

**Keywords:** global eye health, challenges, cataract, refractive error, solutions

As the landscape of vision-threatening diseases continues to evolve with changing lifestyles and population aging, we must adapt our responses and strategies accordingly in a timely manner (1). Currently, age-related and metabolic eye diseases are on the rise, and adolescent refractive errors continue to be a significant concern (Yin et al.). Additionally, the global burden of cataracts and cataract-related blindness, particularly in low-income communities, remains a pressing issue. Therefore, in this Research Topic, we focus on research articles, systematic reviews, and analyses of the epidemiology, prediction, intervention, and policy of disabling eye diseases. By enhancing our understanding of these factors, we hope to inform better development of strategies for preventing vision loss, reducing the prevalence, incidence rate, and progression of eye diseases and ultimately improving both visual quality and quality of life for individuals worldwide.

Cataract is the leading cause of visual impairment and blindness in the older adults. Wang et al. used data from the Global Burden of Disease study to analyze the global distribution and trends in the burden of cataracts in 204 countries and territories from 1990 to 2019. The article proposes that reducing particulate matter pollution, quitting smoking, controlling blood glucose, and lowering BMI can reduce the risk of cataracts. It was found that the age-standardized disability-adjusted life years (DALYs) and mortality caused by each risk factor were highest in the low-middle sociodemographic index (SDI) region, and the overall disease burden of cataracts is lower in males than in females.

Additionally, Lange et al. found that although cataract surgery rates have risen dramatically in the last decade, access to care remains unequal. Low-income patients and women are less likely to receive surgical treatment for cataracts. The lack of funding remains a significant barrier. Ahluwalia et al. also found that the cost of an eye exam can be an obstacle to routine eye care, with the most common reason for not seeking eye care among those with moderate-to-severe visual impairment being cost or lack of insurance. Providing free comprehensive eye examinations and prescription eyeglasses to vulnerable populations, such as homeless individuals and those living in poverty, in the free eye clinic located inside a homeless shelter may be one way to solve the funding shortage.

With the prevalence of myopia increasing sharply over the past decades, preventing visual impairment caused by pathological myopic fundus is a major public health issue. Liu et al. found from a study based on the Genome-Wide Association Study (GWAS) database that genetically predicted taller height, longer time on the computer, and less moderate physical activity are risk factors of myopia. After full adjustment for confounders, height still remained independently and significantly associated with myopia. While a study by Zhou et al. found no significant change in myopia prevalence among senior students in eastern China before and during the COVID-19 epidemic, the prevalence of myopia among vocational high school students is lower compared with general high schools. The effect of educational stress on the prevalence of myopia among students should be paid attention. These findings provide a theoretical basis for future measures to prevent and control myopia in adolescents.

In addition to cataract and refractive error, this issue also discusses ocular trauma. Chen et al. described the epidemiological characteristics of open eye injuries in a multiethnic region of Southwest China. It showed that the type of injury varied by age and occupation, and the cause of injury was related to age, ethnicity, and profession. Ocular trauma disproportionately affected working-aged males, particularly those engaged in manual labor or farming. Sharp objects were identified as the most common cause of these injuries. To effectively reduce the incidence and severity of ocular trauma, it is essential to increase public awareness of ocular trauma prevention and develop personalized prevention and control plans for different population groups.

Majithia et al. discovered a significant correlation between the prevalence of cardiovascular diseases and thinner average peripapillary retinal fiber layer (RNFL) in the multi-ethnic Asian population in Singapore. This association was consistent for the superior and inferior RNFL quadrants. However, cardiovascular diseases were not significantly associated with average macular ganglion cell-inner plexiform layer thickness. This provides a new predictor for cardiovascular diseases. Gao et al. proposed that the reduction of vascular density (VD) in the deep vascular complex seems to be involved in or be accompanied by non-exudative macular neovasculogenesis (MNV) activation. VD could be used as a biomarker for non-exudative MNV, potentially aiding in the early detection and prevention of delayed treatment or overtreatment of subclinical MNV.

Yin et al. conducted a retrospective analysis of trends in the global burden of vision loss among old adults from 1990 to 2019. The prevalence increased and the years lived with disability (YLDs)decreased among individuals aged 65 years and older. The research explored various factors, including the specific types of vision loss, different age groups, geographical regions, countries, and sociodemographic index. Females had higher global prevalence and YLDs compared with males. Cataracts and myopia emerged as the primary drivers of vision loss. Their findings shed light on the complexities of vision loss in the geriatric population and underscore potential areas for targeted interventions across different demographics.

The escalating demand for vision care services, driven by demographic shifts and lifestyle changes, has exacerbated the challenges facing eye health. To effectively address these challenges, we must integrate vision care into broader healthcare strategies, emphasize the importance of preventive measures and available corrective solutions, enhance public awareness of eye health knowledge, strengthen screening and management of eye diseases and their risk factors, and improve the inclusiveness and quality of eye care services.
